# The role of the endolysosomal pathway in α-synuclein pathogenesis in Parkinson’s disease

**DOI:** 10.3389/fncel.2022.1081426

**Published:** 2023-01-10

**Authors:** Jessica K. Smith, George D. Mellick, Alex M. Sykes

**Affiliations:** Griffith Institute for Drug Discovery, Griffith University, Nathan, QLD, Australia

**Keywords:** Parkinson’s disease, α-synuclein, endolysosomal, endocytosis, trafficking

## Abstract

Parkinson’s disease (PD) is a chronic neurodegenerative disease that is characterized by a loss of dopaminergic neurons in the substantia nigra pars compacta of the midbrain (SNpc). Extensive studies into genetic and cellular models of PD implicate protein trafficking as a prominent contributor to the death of these dopaminergic neurons. Considerable evidence also suggests the involvement of α-synuclein as a central component of the characteristic cell death in PD and it is a major structural constituent of proteinaceous inclusion bodies (Lewy bodies; LB). α-synuclein research has been a vital part of PD research in recent years, with newly discovered evidence suggesting that α-synuclein can propagate through the brain via prion-like mechanisms. Healthy cells can internalize toxic α-synuclein species and seed endogenous α-synuclein to form large, pathogenic aggregates and form LBs. A better understanding of how α-synuclein can propagate, enter and be cleared from the cell is vital for therapeutic strategies.

## 1. Parkinson’s disease

Parkinson’s disease (PD) is the second most common neurodegenerative disorder worldwide, with approximately 1% of individuals over 60 years of age manifesting the disease (de Lau and Breteler, 2006; DeMaagd and Philip, 2015; Pang et al., 2019). PD is a multifactorial disease generally with a late onset, as the most prominent risk factor for PD is age (Dauer and Przedborski, 2003; DeMaagd and Philip, 2015; Pang et al., 2019). PD is characterized by the progressive death of dopaminergic neurons in the substantia nigra pars compacta of the midbrain (SNpc) (DeMaagd and Philip, 2015; Deng et al., 2018). Loss of dopamine, a vital neurotransmitter, causes the commonly associated motor symptoms observed in PD patients (Dauer and Przedborski, 2003; DeMaagd and Philip, 2015; Pang et al., 2019). However, by the stage of diagnosis, approximately 80% of dopaminergic neurons are lost in the SNpc (Wu et al., 2019). In the SNpc of post mortem PD brains, dopaminergic neurons commonly contain proteinaceous inclusions known as Lewy bodies (LBs), either believed to cause neuronal death or be a protective measure produced by the neurons themselves (Spillantini et al., 1997, 1998; Beach et al., 2009; Beyer et al., 2009). LBs are composed of misfolded and ubiquitinated proteins that have accumulated over time (Spillantini et al., 1998; Beyer et al., 2009; Shahmoradian et al., 2019). Therefore, dysfunction of protein trafficking has been a widely attributed cause of PD and forms the basis of ongoing research (Spillantini et al., 1998; Beyer et al., 2009).

Lewy bodies are intracytoplasmic inclusions containing primarily aggregated α-synuclein (Spillantini et al., 1998; Mahul-Mellier et al., 2020). Recent studies using correlative light electron microscopy have observed an abundance of crowded membranous material in LBs including lipids, fragmented vesicles, lysosomes and mitochondria (Shahmoradian et al., 2019). Lewy neurites are precursors of LBs and contain predominantly misfolded α-synuclein (Braak et al., 1999). There are two types of LBs that have been defined: brainstem and cortical (Beyer et al., 2009). Brainstem LBs are intracytoplasmic, eosinophilic masses that possess a dense core (Spillantini et al., 1998; Beyer et al., 2009; Tofaris et al., 2017). Cortical LBs are eosinophilic, irregular in shape and poorly defined structures without a central core (Spillantini et al., 1998; Beyer et al., 2009). Importantly, both forms of LBs are composed of filamentous structures. LBs are widely distributed in the central nervous system (CNS) and have been observed in the hypothalamus, SNpc and within cerebrospinal fluid of the spinal cord (Volpicelli-Daley et al., 2014). This suggests that LBs may form and deposit in a variety of areas in the CNS or have the ability to propagate (Volpicelli-Daley et al., 2014). Furthermore, Lewy pathology is also evident in the enteric nervous system and can manifest gastric symptoms in PD patients (Chalazonitis and Rao, 2018). The widespread distribution of LB pathology corresponds to the variety of motor and non-motors symptoms in PD, as the deposition can cause various deficits, such as loss of olfaction (Beach et al., 2009; Beyer et al., 2009; Luk et al., 2009).

The etiology of PD results from an elaborate interplay of genetics, environmental exposures, gene-environment interactions and the direct impact of these factors on the aging brain (Klein and Westenberger, 2012). Underlying causes and stressors continue to be identified, often through genetic screening of familial PD patients.

### 1.1. Genetics of Parkinson’s disease

Roughly 5% of PD cases can be attributed to a single genetic mutation (monogenic) (Deng et al., 2018). The first genetic mapping of PD identified a mutation in *SNCA*, responsible for the pathogenesis leading to manifestation of parkinsonian symptoms (Polymeropoulos et al., 1997). The complexity of genetic causes for PD continues. In the 20 years since the identification of *SNCA* (the first PD gene) there are 23 distinct chromosomal regions that are related to a genetic form of PD (Lesage and Brice, 2009; Day and Mullin, 2021). Interestingly, a vast number of identified PD mutations are involved in endolysosomal sorting of proteins, including α-synuclein.

### 1.2. PD mutations associated with protein trafficking

Parkinson’s disease mutations have elucidated the molecular mechanisms and vital pathways that link genes of interest to pathogenesis of the disease (Schulte and Gasser, 2011; Day and Mullin, 2021). Therefore, understanding how genes that result in parkinsonian symptoms are involved with the trafficking of the major component of LBs, α-synuclein is vital to further understanding how proteins are trafficked in people living with PD. Although monogenic forms of disease only account for a small number of cases, sporadic PD can be caused by the same cellular dysfunction. Furthermore, elucidating these vital pathways of α-synuclein trafficking with the involvement of genetic mutations can provide avenues for future drug screening.

#### 1.2.1. *SNCA*

*SNCA* was the first genetic mutation causally associated with autosomal-dominant PD (Polymeropoulos et al., 1997). *SNCA* mutations are rare in the general population, with five presumably causal point mutations now discovered (Table 1). Duplications and triplications of the entire gene have also been previously reported in familial cases of PD (Singleton et al., 2003). Through these genetic studies it was discovered that overexpression of α-synuclein could produce aggregates and potentially lead to Lewy pathology (Singleton et al., 2003). *SNCA* variants demonstrate high penetrance, with 85% of people with the most common A53T variant manifesting the disease (Klein and Westenberger, 2012). Five missense mutations associated with PD (Table 1) reside in the amino-terminal domain, demonstrating importance within the region for pathogenicity of the protein (Polymeropoulos et al., 1997; Krüger et al., 1998; Zarranz et al., 2003; Proukakis et al., 2013; Pasanen et al., 2014; Kiely et al., 2015). These single nucleotide variants tend to form the stable β sheets that skew the protein toward irreversible aggregation and exacerbate the formation of toxic oligomers and fibrils (Stefanis, 2012; Flagmeier et al., 2016).

**TABLE 1:** *SNCA* single nucleotide variants linked to PD pathogenesis.

Mutation	Protein domain	Inheritance	References
A30P	Amphipathic	Autosomal dominant	Krüger et al., 1998
E46K	Amphipathic	Autosomal dominant	Zarranz et al., 2003
H50Q	Amphipathic	Autosomal dominant	Kiely et al., 2015
G51D	Amphipathic	Autosomal dominant	Proukakis et al., 2013; Kiely et al., 2015
A53T/E	Amphipathic	Autosomal dominant	Polymeropoulos et al., 1997; Pasanen et al., 2014

There are five identified missense mutations in SNCA that are rare, autosomal dominant inherited forms of Parkinson’s disease.

#### 1.2.2. *VPS35*

In 2011, a missense mutation in vacuolar protein sorting homolog 35 (VPS35) was linked to autosomal dominant PD (Zimprich et al., 2011). VPS35 is a vital portion of the retromer complex that mediates transport of cargo from the endosomal system to the trans-Golgi network (Zimprich et al., 2011). VPS35 is part of the cargo recognition portion of the retromer (Zimprich et al., 2011). The retromer complex is responsible for delivery of lysosomal enzymes, such as cathepsin D, to lysosomes (Miura et al., 2014). Cathepsin D is a soluble aspartic endopeptidase involved in the protein degradation in the strongly acidic milieu of lysosomes (Kollmann et al., 2013). Cathepsin D is also known as one specific enzyme that can breakdown α-synuclein (Sevlever et al., 2008). Therefore, dysfunction to the transport of cathepsin D via mutations in the retromer complex can lead to an alteration in degradation of α-synuclein (Sevlever et al., 2008). When VPS35 was silenced in *Drosophilla*, intracellular α-synuclein accumulated in the late endolysosomal compartments, indicating the retromer complex plays a critical role in α-synuclein catabolism (Miura et al., 2014).

#### 1.2.3. *LRRK2*

Mutations in leucine-rich repeat kinase 2 (LRRK2) are the most frequent causes of both autosomal-dominant and sporadic PD with more than 50 different missense mutations reported and at least 16 have been associated with pathogenicity (Cilia et al., 2014; Deng et al., 2018). *LRRK2* encodes a complex multi-domain 2527 amino acid cytoplasmic protein, LRRK2 (Deng et al., 2018). G2019S is the most common and studied variant and accounts for as many as 40% of cases in certain geographic denominations, such as the North African Berber population (Lesage and Brice, 2009; Cilia et al., 2014; El Haj et al., 2017). LRRK2 and α-synuclein have been seen to physically interact and can directly regulate the function and/or activity of the other (O’Hara et al., 2020). α-synuclein is either present natively in the cytosol or vesicular structures of neurons that is pathogenic when phosphorylated (O’Hara et al., 2020). LRRK2 is a serine-threonine kinase that was thought to directly phosphorylate α-synuclein in neurons which can result in aggregation and Lewy body formation (Zimprich et al., 2004; O’Hara et al., 2020). *In vitro* studies have since demonstrated that the G2019S mutant can induce an indirect kinase-dependent increase in levels of phosphorylated α-synuclein, demonstrated to be a pathogenic posttranslational modification to the protein (Guerreiro et al., 2012). Other studies have further sought to investigate the relationship between α-synuclein and LRRK2 and suggest that LRRK2 could regulate the cell-to-cell transmission of α-synuclein and interrupt autolysosomal degradation of α-synuclein (O’Hara et al., 2020).

#### 1.2.4. *GBA*

Lysosomes are vital organelles involved in synucleinopathies and are the main degradative component of the cell (Ferreira and Gahl, 2017). Defects in lysosomal function can result in lysosomal storage disorders and often characterize progressive neurodegenerative diseases, such as PD (Ferreira and Gahl, 2017). The most common lysosomal storage disorder is Gaucher disease which results from the loss of function of lysosomal enzyme β-glucocerebrosidase (GCase) (Ferreira and Gahl, 2017). Individuals that manifest Gaucher disease as well as carriers of heterozygous mutations in *GBA* are at increased risk of developing PD due to the dysfunctional lysosomes (Ferreira and Gahl, 2017). Dysfunction of GCase in the lysosomes can increase α-synuclein accumulation directly, causing excess of misfolded proteins within the cell (Bae et al., 2014). Functional loss of GCase causes the accumulation of glucocerebroside, which directly influences aggregation of α-synuclein as it can stabilize the oligomeric intermediates that are thought to be the most toxic form of α-synuclein (Bae et al., 2014, 2015; Mazzulli et al., 2016). Therefore, this creates a positive feedback loop of α-synuclein and β-GCase that leads to accumulation of α-synuclein (Mazzulli et al., 2016).

#### 1.2.5. *ATP13A2*

Adenosine triphosphate cation transporting 13A2 (ATP13A2) encodes an integral decamembrane spanning ATPase located in the lysosomal membrane that functions as a late endo-/lysosomal polyamine transporter (van Veen et al., 2020). Familial *ATP13A2* mutations were demonstrated to enhance α-synuclein aggregation and promote cell death (Si et al., 2021). Research indicates that ATP13A2 and α-synuclein interact in the endolysosomal system and that ATP13A2 directly affects α-synuclein homeostasis (Fonseca et al., 2015). Loss of ATP13A2 impairs lysosomal membrane integrity and induces α-synuclein mutlimerisation at the ER membrane which then can further the pathology of α-synuclein (Fonseca et al., 2015). Further, ATP13A2 deficiency in neuroblastoma cells leads to lysosomal dysfunction and reduces the function of the endolysosomal trafficking of α-synuclein (Si et al., 2021).

These genetic studies illustrate the importance of the homeostasis of vesicular trafficking pathways as dysregulation is highly correlated with the progression of PD. Furthermore, variants of proteins involved in the endolysosomal pathway are emerging as important for their involvement in PD.

### 1.3. Cellular dysfunction in PD

**FIGURE 1:**
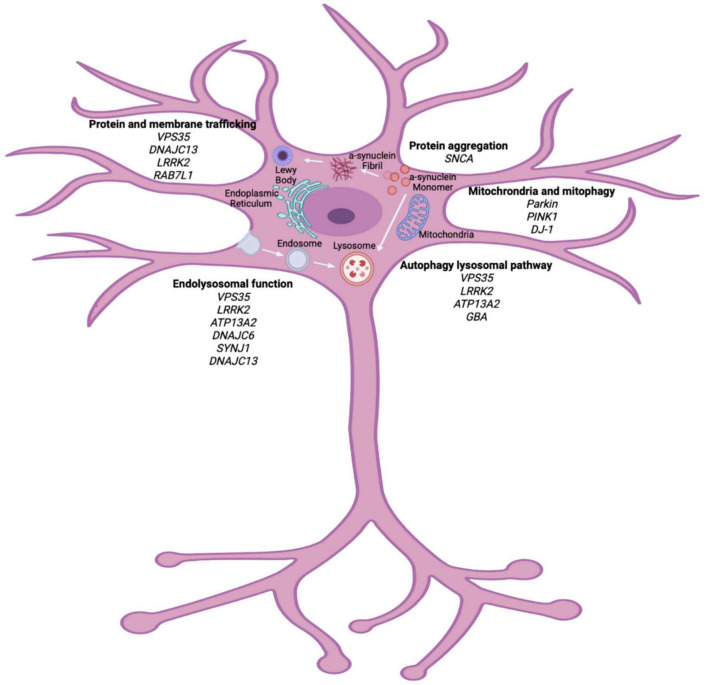
Cellular pathways implicated in PD pathogenesis. Genetic studies of heritable PD have highlighted vital pathways that can contribute to the pathogenesis of PD. Organelle dysfunction within the neuron, due to many genetic and environmental factors, can lead to cellular stress and eventual neuronal death. Major pathways outline here implicate protein trafficking, endolysosomal dysfunction, autophagy lysosomal pathway and mitochondrial function as vital in PD pathogenesis. Biorender image (adapted from Polymeropoulos et al., 1997; Zimprich et al., 2011; Krebs et al., 2013; Macleod et al., 2013; Vilariño-Güell et al., 2013; Cilia et al., 2014; Fonseca et al., 2015; Ferreira and Gahl, 2017).

A combination of environmental and genetic factors are generally thought to contribute to the development of PD; however, it is believed that cellular stress precipitates neuronal cell death in the SNpc (Levy et al., 2009). Currently, the pathogenic mechanisms of interest include mitochondrial dysfunction, oxidative stress and protein misfolding that can be caused by genetic mutations (Figure 1). Understanding the pathways of cellular dysfunction will aid in identifying biomarkers and better treatment options.

#### 1.3.1. Endolysosomal dysfunction and autophagy

The endolysosomal pathway is the main route for uptake, processing and clearance of cargoes collected from the extracellular space (Vidyadhara et al., 2019). It comprises a highly dynamic network of vesicular structures that facilitates nutritional intake, autophagy and maintenance of cellular homeostasis (Vidyadhara et al., 2019). The major endocytic route for internalization of many cargoes and the primary route of uptake in neurons is clathrin-mediated endocytosis (Robinson, 1994; Ford et al., 2002; Delenclos et al., 2017). This specific process is initiated when endocytic coat proteins from the cytosol cluster on the inner leaflet of the plasma membrane (Robinson, 1994; Ford et al., 2002; Delenclos et al., 2017). The assembling coat promotes positive membrane curvature and transforms into a clathrin-coated pit (Robinson, 1994; Ford et al., 2002; Vidyadhara et al., 2019). Upon entering the cytoplasm, primary endocytic vesicles undergo homotypic fusion and become early/sorting endosomes (Delenclos et al., 2017). Cargoes can then be recycled to the plasma membrane, sent to the trans-Golgi network or sorted to the lysosome for degradation (Robinson, 1994; Ford et al., 2002; Delenclos et al., 2017). Increasing evidence suggests that the endolysosomal system is the key pathway affected in PD (Kett and Dauer, 2016). The role of the endolysosomal system in PD has been elucidated through genetic studies including genome wide association studies which identified a large portion of disease-associated risk genes belonging to the endolysosomal system (Nalls et al., 2019). *DNAJC6* encodes for auxilin a major pre-synaptic endocytic protein that is a chaperone and plays an important role in clathrin uncoating (Köroğlu et al., 2013). Mutations in *DNAJC6* have been linked to PD, suggesting a vital role for endocytic processing in pathogenesis of disease. Transmembrane protein 106B *(TMEM106B)* encodes for TMEM106B, a lysosomal membrane protein which is highly expressed in CNS neurons and is localized to late endolysosomal compartments (Brady et al., 2013; Feng et al., 2021). TMEM106B plays a vital role in lysosomal function, where overexpression demonstrated lysosomal enlargement, oxidative stress, and subsequent cell death in HeLa cells (Lang et al., 2012; Brady et al., 2013; Suzuki and Matsuoka, 2016; Feng et al., 2021). In recent years, *TMEM106B* has been linked to many neurological disorders and genome wide association studies has recently found that *TMEM106B* may play a role in PD, demonstrating a vital role for endolysosomal processing in PD (Tropea et al., 2019).

Autophagy is a key component of the endolysosomal system and is an intracellular protein clearance pathway to maintain cellular homeostasis (Demirsoy et al., 2017). Lysosomes are organelles that contain proteolytic enzymes that play a role in proteostasis and are the primary destination for a large portion of endolysosomal system cargo (Demirsoy et al., 2017). Lysosomal dysfunction can perturb cellular homeostasis (Bento et al., 2016). Mutations in *ATP13A2* has shown impaired lysosomal acidification leading to dysfunctional protein degradation and autophagosome clearance (Bento et al., 2016). One of the most well-defined forms of monogenic PD results from point mutations in the *LRRK2* mutant and it has been discovered to have a role in lysosomal homeostasis and autophagy (Kett and Dauer, 2016; Kuwahara and Iwatsubo, 2020). Phosphorylation by LRRK2 is essential for a subset of guanosine triphosphatases (GTPases) that function to regulate endosomal trafficking (Kett and Dauer, 2016; Kuwahara and Iwatsubo, 2020). Mutations in *LRRK2* significantly affect Ras-associated binding proteins (Rab) and are strongly related to PD (Kett and Dauer, 2016; Kuwahara and Iwatsubo, 2020). Protein uptake, trafficking and clearance is therefore a vital pathway to understand and investigate in PD pathogenesis and is a key target for therapeutic intervention (Vidyadhara et al., 2019).

New treatment options could be aimed at earlier interventions targeting cellular dysfunction prior to neuron loss. Protein trafficking is one of the overarching cellular dysfunction mechanisms in PD (Beyer et al., 2009). A common theme in recent PD research is identifying ways to prevent or alter progression of PD through α-synuclein manipulation. Therefore, α-synuclein aggregation has been widely targeted for treatment of PD with varying success.

## 2. α-synuclein

### 2.1. Physical chemistry

α-synuclein, a 14 kDa cytoplasmic protein, is encoded by the gene *SNCA* is one of the most abundant proteins found in the nervous system constituting ∼1% of cytosolic protein (Maroteaux and Scheller, 1991; Kahle, 2007; Stefanis, 2012). α-synuclein was first discovered in 1988 when it was purified from cholinergic synaptic vesicles and was found to contain a highly conserved core region (Maroteaux et al., 1988). α-synuclein was initially believed to only be expressed in nervous system tissue, however, it is now known to be widely expressed in the human body (Maroteaux et al., 1988; Stefanis, 2012). In the brain, α-synuclein is enriched in pre-synaptic terminals and functions in synaptic vesicle transmission (Maroteaux and Scheller, 1991; Bellani et al., 2010; Burré et al., 2012). The biochemical properties of α-synuclein resemble a chaperone as its domains are capable of binding to other proteins, particularly lipid-rich domains. α-synuclein has an 11-mer repeat that recurs seven times (Burré et al., 2018). The soluble protein exists in a random, open conformation, which promotes its binding. The 11-mer repeat has been shown to promote binding to phospholipid vesicles (Bisalgia et al., 2006; Burré et al., 2018).

The primary structure of the protein contains three regions. The N-terminal region (residues 1–60) contains repetitions of highly conserved lysine repeats (Bisalgia et al., 2006; Burré et al., 2012, 2018). The N-terminal domain is amphipathic which allows for interactions with membranes and serve the lipid-binding function of the protein (Bisalgia et al., 2006; Burré et al., 2012, 2018). The N-terminal region has α-helical propensity that resembles that of apolipoprotein-binding domains. The hydrophobic region (residues 61–95) which is known as the non-amyloid β component (NAC) domain is required for amyloid formation and has the ability to aggregate independent of the protein (Bisalgia et al., 2006; Burré et al., 2012, 2018). The NAC motif mediates conformation changes of α-synuclein to form β-sheets (Bisalgia et al., 2006; Burré et al., 2012, 2018). Finally, the acidic C-terminal region (residues 96–140) is believed to shield the NAC domain from aggregating spontaneously (Meuvis et al., 2010). Deletion of any portion of the C-terminus can accelerate α-synuclein aggregation *in vitro* (Meuvis et al., 2010). The C-terminal region does not contain rigid secondary structure; however, it does interact with other domains of the protein to remain natively unfolded (Meuvis et al., 2010).

### 2.2. Function

Originally, α-synuclein was considered as an intrinsically disordered protein that cycled between natively unfolded state in the cytosol and folded on lipid membranes, whereby the function of the protein was not yet elucidated (Bonini and Giasson, 2005). Increasing evidence suggests that α-synuclein plays a role in synaptic vesicle trafficking and neurotransmitter release at the synapse (Burré et al., 2010). α-synuclein can promote assembly of the soluble N-ethylmaleimide-sensitive factor attach protein receptor (SNARE) complex that mediates synaptic vesicle fusion, which is a primary step in neurotransmitter release (Burré et al., 2010; Hawk et al., 2019). α-synuclein can act as a chaperone to the SNARE complex (Burré et al., 2010). Early dysfunction of α-synuclein can therefore trigger impaired neurotransmission, including dopamine release, and synaptic dystrophy (Hawk et al., 2019). Munc-18-1, an essential component of the molecular machinery, controls SNARE membrane fusion in neurons has been reported as a chaperone for α-synuclein and can control its aggregative propensity (Chai et al., 2016).

### 2.3. Pathogenesis

The pathogenesis of α-synuclein provides direction for drug discovery, as the conformation of the protein and how it evades cellular degradation can alter drug screening techniques. As described, α-synuclein was first thought to be pathogenic when the *SNCA* gene was linked to autosomal dominant forms of PD; early genetic research also demonstrated that duplications and triplications of the *SNCA* gene could also be associated with disease state (Polymeropoulos et al., 1997; Singleton et al., 2003). Hence, gene dosage and increased protein expression is related to synuclein pathology in the brain (Singleton et al., 2003; Stefanis, 2012). These findings highlighted the generic mechanisms by which α-synuclein could become pathogenic: increased protein levels and point mutations that exacerbate aggregation (Polymeropoulos et al., 1997; Singleton et al., 2003; Stefanis, 2012). The link between α-synuclein and PD continued to be strengthened when phosphorylated and aggregated α-synuclein was found to be the primary component of proteinaceous LBs (Spillantini et al., 1997, 1998; Beyer et al., 2009). Due to the genetic findings, researchers were led to develop antibodies against α-synuclein to use in histopathological sections of PD patient brains, discovering that α-synuclein was robustly expressed in LBs in the halo-like inclusions (Spillantini et al., 1998).

The ability of α-synuclein to generate β sheets provided parallels to β-amyloid and unified the pathogenic basis between the two most common neurodegenerative diseases Alzheimer’s disease and PD (Lashuel et al., 2013). In the context of PD, both α-synuclein and α-synuclein*A*^53T^ form amyloid structures upon prolonged incubation in solution, however, α-synuclein*A*^53T^ has increased aggregation kinetics (Conway et al., 2001; Stefanis, 2012; Lashuel et al., 2013). α-synuclein adopts conformations that allows the establishment into stable seeds that can act as a conformational template of the amyloid state (Conway et al., 2001; Courte et al., 2020; Mahul-Mellier et al., 2020). The aggregation of α-synuclein into amyloid fibrils is a multi-step process that conducts various intermediate states (Conway et al., 2001; Courte et al., 2020). The current dogma of α-synuclein aggregation suggests that upon initial incubation of natively unfolded and soluble monomers, soluble oligomeric forms of α-synuclein are formed that can assume spherical-like structures when visualized under electron microscopy (Pieri et al., 2016). The various forms of oligomers are termed protofibrils and gradually aggregate to become insoluble fibrillar structures (Pieri et al., 2016). *In situ*, α-synuclein only requires agitation to aggregate, however, in the brain it is environmental exposures, genetic mutations and cellular stress that can drive the conformational shift to larger, misfolded multimeric states (Volpicelli-Daley et al., 2011). It is thought that α-synuclein may become pathogenic *via* different non-mutually exclusive mechanisms. These include the formation of insoluble α-synuclein aggregates causing cell death and the propagation of the protein *via* spreading and seeding endogenous monomers to aggregate (Stephens et al., 2020). To understand how aggregation is started, researchers slowly induced aggregation using calcium ions, a known stimulator of aggregation (Stephens et al., 2020). The N-terminus of the protein can unfold upon exposure to calcium and the NAC region can be exposed to the environment becoming more aggregation prone (Stephens et al., 2020). The level of exposure and post translational modifications are highly determinant on rate and propensity of aggregation (Stephens et al., 2020).

The function of α-synuclein at the synapse resulted in significant synaptic deficits upon α-synuclein misfolding and aggregation (Nakamura et al., 2011). Induced neurotoxic *in vitro* and *in vivo* models based on overexpression of the protein has seen loss of neurotransmitter release, redistribution of SNARE proteins and inhibition of vesicle recycling (Nakamura et al., 2011; Stefanis, 2012).

α-synuclein is subject to several post-translational modifications that can also impact pathogenesis including N-terminal acetylation, ubiquitylation, SUMOylation, nitration, and phosphorylation (Zhang et al., 2019). The most studied α-synuclein posttranslational modification is phosphorylation of serine 129 which results in higher susceptibility to aggregation (Fujiwara et al., 2002; Zhang et al., 2019). Furthermore, LBs contain primarily phosphorylated α-synuclein, suggesting a potentially pathogenic role for this posttranslational modification (Spillantini et al., 1997, 1998; Fujiwara et al., 2002). Studies have shown that phosphorylated α-synuclein can induce unfolded protein response-mediated cell death in neuronal-like cells (Sugeno et al., 2008).

**FIGURE 2:**
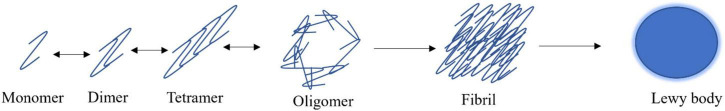
α-synuclein aggregation model. α-synuclein exists as natively unfolded monomers that can reversibly form dimers, tetramers, and oligomeric species. α-synuclein has the propensity to aggregate irreversibly into β-rich fibrils which are the primary component of Lewy bodies.

The dogma of α-synuclein aggregation begins with natively unfolded monomers reversibly forming higher molecular weight oligomers that can assume spherical-like structures (Figure 2; Fauvet et al., 2012; Lashuel et al., 2013; Ghosh et al., 2017). The various forms of oligomers can gradually aggregate to become insoluble fibrils that can accumulate into proteinaceous inclusions. Smaller, soluble species of α-synuclein can be broken down by the proteasome, where larger species require the autophagolysosomal pathway for degradation (Figure 2; Menezes et al., 2015). After α-synuclein has formed large, fibrillar structures, it can accumulate into LBs (Figure 2; Wakabayashi et al., 2007; Volpicelli-Daley et al., 2011; Ghosh et al., 2017). α-synuclein is involved in the earliest stages of LB formation and continues to accumulate as the LB progresses (Wakabayashi et al., 2007; Volpicelli-Daley et al., 2011; Ghosh et al., 2017). Further, α-synuclein aggregates are seen more in dopaminergic neurons of the substantia nigra in PD patients than LBs (Zhang et al., 2019). The other major components of LBs are all α-synuclein-binding proteins including agrin, 14-3-3, MAP1B, synphilin-1, and tau (Jensen et al., 2000; Wakabayashi et al., 2007). It is proposed that α-synuclein, the largest component of LBs, recruits its binding proteins to further the pathology of these aggregates (Wakabayashi et al., 2007).

### 2.4. Conformations of α-synuclein

#### 2.4.1. Monomers

Monomers of α-synuclein are typically characterized as 14 kDa, soluble and intrinsically disordered as these species do not have a stable conformational state (Maroteaux and Scheller, 1991; Kahle, 2007; Ferreon et al., 2009; Stefanis, 2012). Monomers are believed to be the predominant conformational species existing in neuronal cytosol (Ferreon et al., 2009; Alam et al., 2019). Monomeric α-synuclein is dynamic and complex and exists in a variety of conformational states, hence why it is considered natively unfolded (Ferreon et al., 2009). α-synuclein conformational plasticity is limited by its functional aspects of lipid binding and membrane curvature for vesicle fusion (Frimpong et al., 2010). Each conformation α-synuclein can adopt depends on interactions by hydrogen bonds, pH and solvent conditions (Alam et al., 2019).

#### 2.4.2. Oligomers

There are an extremely large variety of early, prefibrillar α-synuclein species called oligomers that differ in molecular weight and structure (Fauvet et al., 2012; Lashuel et al., 2013; Pieri et al., 2016; Ghosh et al., 2017). Whether oligomers or fibrils are the most toxic species of α-synuclein is still debated widely in literature. Oligomers have the ability to spread and can potentiate into fibrils at any part of the cell, making them pathogenic (Winner et al., 2011; Fauvet et al., 2012; Ghosh et al., 2017). Overexpression of α-synuclein oligomers can lead to cellular toxicity *in vitro* (Winner et al., 2011). Oligomeric species can impair the autophagy lysosomal pathway and ubiquitin proteasome system protein degradation system, disrupt lipid bilayer and increase the influx of calcium ions to increase aggregation, cause cytoskeletal alternations, damage mitochondrial and endoplasmic reticulum membranes and increase ROS production (Danzer et al., 2007; Parihar et al., 2009; Colla et al., 2012).

There are two main oligomeric subsets: on-fibrillar assembly pathway and off-fibrillar assembly pathway (Alam et al., 2019). However, this is complicated as both types of oligomers present in many shapes and sizes and ensues differences in reactivity and toxicity (Winner et al., 2011). On-pathway fibrillar oligomers are species that directly form or take part in fibril formation (Alam et al., 2019). Oligomers that exhibit a degree of stability and do not tend toward fibrillization are off-fibrillar species (Alam et al., 2019). These are stable species that can be formed in the early stages of fibrillation (starting with monomers) that do not eventuate into fibrils. Off-pathway oligomers can be formed by addition of small molecules, such as epigallocatechin gallate (EGCG) that inhibit fibrillization but not oligomerization (Alam et al., 2019).

#### 2.4.3. Fibrils

The fibrillar form of α-synuclein is most commonly associated with PD as it is located within LBs and inside the neuronal cytoplasm (Spillantini et al., 1998; Volpicelli-Daley et al., 2011; Stefanis, 2012; Ghosh et al., 2017; Gómez-Benito et al., 2020). Both toxic and non-toxic α-synuclein fibrillar species have been reported; these have varying effects on neuron survival (Alam et al., 2019). The fibrils that have been proposed to contribute to neurodegeneration are those that seed assembly of soluble α-synuclein species to form high molecular weight aggregates and form an imbalance in cellular proteostasis (Volpicelli-Daley et al., 2011; Alam et al., 2019). Fibrillar species are believed to be toxic due to their propagation, amplification, and seeding properties (Danzer et al., 2012). This is the clearest distinction of function between the two-toxic species. On-pathway oligomers have been shown to possess seeding activities but not to the length and pathogenicity of fibrils (Alam et al., 2019).

Kinetics of α-synuclein fibril formation demonstrate a lag phase (nucleation phase), an exponential phase (elongation) and a plateau phase that demonstrates fibrils formation (Arosio et al., 2015; Iljina et al., 2016). This occurs over 7 days of monomers shaking at 37°C (Li et al., 2018). Furthermore, fibrillization of α-synuclein is irreversible, which is a key determinant against reversible and soluble oligomeric species (Tuttle et al., 2016). Nuclear magnetic resonance spectroscopy has deduced a “Greek key” conformation where each α-synuclein subunit within the fibril has a β-sheet conformation with hydrogen bonding between adjacent α-synuclein subunits spaced evenly apart (Tuttle et al., 2016). The central β-sheet core is located primarily within the NAC region (Tuttle et al., 2016). N- and C- termini display flexible, random coil that is poorly reserved in the structure (Tuttle et al., 2016). Electron microscopy has shown typical fibrils range from 10 nm in diameter, comparable to the PD brain (Meade et al., 2019; Yang et al., 2022). Approximately 25% of extracted α-synuclein filaments from the cingulate cortex of post-mortem PD brains contained helical twists which allowed for structural determination. α-synuclein filaments were consistently single protofilaments, whereby the ordered core of the filament was termed a Lewy fold (Yang et al., 2022). A Lewy fold is formed by 31–100 residues which arrange as 9 β-strands that layer within the highly ordered core region (Yang et al., 2022). The Lewy fold differs from other synucleinopathies (such as multiple system atrophy), however, remained consistent across Lewy body diseases (Yang et al., 2022). This finding was consistent with differences in α-synuclein seeding amplification between PD and multiple systems atrophy, suggesting the importance of filament conformation is vital for pathological seeding (Yamasaki et al., 2019; Yang et al., 2022).

### 2.5. Uptake, trafficking and clearance of α-synuclein

**FIGURE 3:**
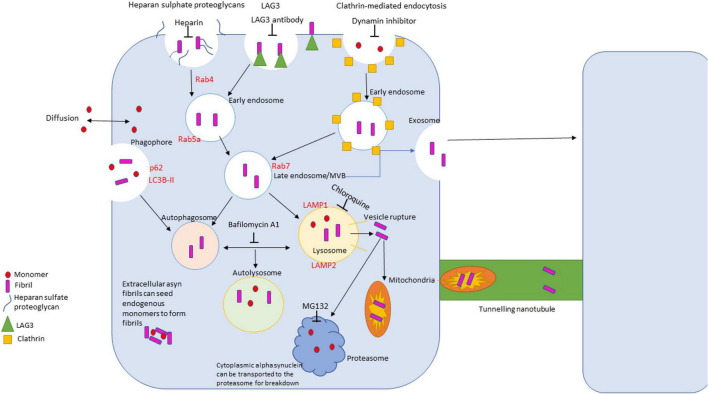
Diagram of uptake, trafficking and clearance mechanisms of α-synuclein in the cell. There are four primary routes of uptake of α-synuclein into the cell: clathrin-mediated, LAG3 receptor-mediated, heparan sulfate proteoglycans-mediated, and passive diffusion. Different conformations of α-synuclein can be internalized via different routes. The autophagolysosomal pathway and the ubiquitin proteasome pathway are the primary clearance mechanisms that degrade α-synuclein. The autophagolysosome pathway can degrade larger species of α-synuclein, where the proteosome breaks down monomers and small, soluble oligomers.

Trafficking of α-synuclein is a complex process that entails different membrane binding proteins, internalization routes and methods of clearance due to the vast difference in conformations of the protein (Figure 3). α-synuclein is constantly secreted into extracellular space and importantly can be taken up by neighboring neuronal cells (Alanko and Ivaska, 2016; Gribaudo et al., 2019). There are a variety of proposed mechanisms for its internalization including: endocytosis, pinocytosis, and cell surface protein-mediated uptake (Alanko and Ivaska, 2016; Gribaudo et al., 2019; Zhang et al., 2020). Understanding these mechanisms is vital to understand α-synuclein pathology due to the protein’s ability to spread throughout the nervous system. The proposed pathogenesis of α-synucleinopathies begins with the cellular impairment regarding protein clearance, leading to aberrant α-synuclein accumulation which induces further proteasomal dysfunction and leads to further degeneration (a vicious cycle) (Stefanis, 2012; Alanko and Ivaska, 2016). α-synuclein has been seen to impact several cellular organelles in models of neurodegeneration including mitochondria, lysosomes, and the endoplasmic reticulum (Gribaudo et al., 2019).

#### 2.5.1. Uptake of α-synuclein

α-synuclein uptake has been widely researched in recent years as it is vital to understand this mechanism for prion spreading pathology and how this process can be inhibited for therapeutic outcomes. All forms of α-synuclein can be efficiently internalized without the need of transfection reagents (Konno et al., 2012; Delenclos et al., 2017; Masaracchia et al., 2018; Rodriguez et al., 2018; Gribaudo et al., 2019). Uptake is cell-type dependent, as astrocytes more readily internalize fibrils than primary neurons (Rodriguez et al., 2018). Within the endocytic pathway, there are several specific- and non-specific routes of uptake and this is the primary pathway that is studied in α-synuclein pathology (Delenclos et al., 2017; Rodriguez et al., 2018).

A commonly used non-invasive inhibitor of endocytosis is low temperature and this can reduce uptake of fibrils in a wide range of non- and neuronal cell lines (Lee et al., 2008). The same effect is seen with dynamin mutants and dynamin inhibitors that block dynamin mediated endocytosis (Lee et al., 2008). Fibrils over 50 nm do not appear to be taken up and there is an upper limit to the uptake pathway (i.e., saturation of the endocytic route) (Rodriguez et al., 2018).

##### 2.5.1.1. Clathrin-mediated endocytosis

The first proposed mechanism of uptake of α-synuclein into cells was clathrin-mediated endocytosis (Lee et al., 2008; Kaksonen and Roux, 2018). Highly conserved mechanisms regulate the dynamic nature of trafficking pathways to control membrane trafficking, such as prolactin induced proteins and Rab (Krishnan et al., 2020). Rab proteins are small GTPases which regulate protein transport and compartmentalization along the endocytic and exocytic pathways (Yap and Winckler, 2009). Studies have suggested that extracellular α-synuclein is internalized via clathrin-mediated endocytosis beginning with α-synuclein accumulating on the plasma membrane (Lee et al., 2008). α-synuclein then enters the cell where it is sorted into Rab4A, Rab5A, and Rab 7 positive vesicles (Masaracchia et al., 2018). Rab7 positive vesicles sorts α-synuclein for degradation via the lysosome and reduces intracellular accumulation of α-synuclein (Masaracchia et al., 2018). α-synuclein has been demonstrated to follow the same route of uptake as transferrin (clathrin-mediated endocytosis) in H4 neuroglioma cell models demonstrating the protein was internalized via the same mechanism (Masaracchia et al., 2018).

Studies using the dynamin inhibitor dynasore and dominant negative dynaminin constructs have demonstrated only partial inhibition of α-synuclein uptake in neural-like cells (Konno et al., 2012; Masaracchia et al., 2018; Gribaudo et al., 2019). This indicates that clathrin mediated endocytosis is not the only route for entry of α-synuclein into a cell. Interestingly, in contrast, a recent study by Choi et al. (2020) showed that in murine microglia, the use of dynasore increased the uptake of α-synuclein (Choi et al., 2020). Overall, there is no general consensus in the literature as to the involvement of clathrin-mediated endocytosis in α-synuclein uptake, as some exclude involvement of clathrin-mediated endocytosis (Kaur and Lee, 2021) whereby others deduce it is vital to internalization (Masaracchia et al., 2018). Clathrin-mediated endocytosis is now believed the main route of uptake for monomers and small, low molecular weight and soluble oligomeric species (Masaracchia et al., 2018). Studies have demonstrated that fibrils can be taken up via clathrin-mediated endocytosis, but largely fibrils enter through other endocytic routes (Tyson et al., 2016).

##### 2.5.1.2. LAG3 receptor

Lymphocyte-activation gene 3 (LAG3; CD223) belongs to the immunoglobulin super family and is expressed by immune cells and neurons (Goldberg and Drake, 2011; Mao et al., 2016). LAG3 is a transmembrane protein that functions primarily to regulate T cell immune responses (Goldberg and Drake, 2011; Mao et al., 2016). Recent evidence highlights the fact that proteins traditionally belonging to the immune system may play critical roles in the CNS and neurological disorders (Mao et al., 2016). Murine neurons were used to investigate the involvement of transmembrane proteins in neuron-to-neuron transmission and found that recombinant mouse α-synuclein fibrils utilize the LAG3 receptor for propagation and found that LAG3 exhibited the highest affinity for fibrils as compared to monomers (Mao et al., 2016). Further examination of the interaction between α-synuclein and LAG3 found the Ig-like domains of LAG3 to bind favorably to α-synuclein fibrillar structures (Mao et al., 2016). Moreover, LAG3 triggered pre-formed fibril (PFF) endocytosis as knockout of the gene significantly reduced internalization of PFFs (Birol et al., 2018). Endocytosis was determined by PFFs residing in Rab5 positive vesicles, suggesting that LAG3 can cause preferential endocytosis of α-synuclein PFFs (Mao et al., 2016; Birol et al., 2018). LAG3 also enhanced the phosphorylation of α-synuclein as serine 129 (Mao et al., 2016). Interestingly, the LAG3 receptor does not bind α-synuclein monomers directly, it preferentially uptakes PFFs and large oligomeric species (Birol et al., 2018).

##### 2.5.1.3. Heparan sulfate proteoglycans

Proteoglycans are glycoproteins that contain sulphated glycosaminoglycan (GAG) chains (Holmes et al., 2013). These chains bind a number of protein ligands and are vital for neuronal function (Holmes et al., 2013). GAGs, particularly heparan sulfate, interact with amyloid proteins. Heparan sulfate proteoglycans (HSPGs) are the primary receptors for macropinocytosis and have been shown to mediate fibrillar tau uptake in Alzheimer’s disease (Holmes et al., 2013). Furthermore, heparan sulfate has been found in extracellular amyloid deposits (Ihse et al., 2017). Studies have shown that in neuronal cells, internalization of α-synuclein in the form of amyloid fibrils depended on heparan sulfate, where soluble monomers and non-amyloid oligomers did not (Ihse et al., 2017). In B103 neuroblastoma cells, heparan sulfate largely co-localized with α-synuclein fibrils pre- and post-uptake (Ihse et al., 2017). This suggests that the interaction between the proteins occurs extracellularly and can then be internalized (Ihse et al., 2017). Blockers of HSPGs include soluble heparan, heparinase, and chloral hydrate; all of which have shown a specific reduction in the uptake of α-synuclein PFFs, but not monomers or oligomers (Holmes et al., 2013; Ihse et al., 2017; Birol et al., 2018).

#### 2.5.2. Diffusion

α-synuclein monomers are reported to passively diffuse in an out of membranes (Lee et al., 2008; Steiner et al., 2018). Due to the small size of the protein and propensity to interact with lipid membranes, it can facilitate passive diffusion in some cell types (Lee et al., 2008). In SH-SY5Y cells, the monomeric protein was internalized and either cleared or exocytosed in approximately 2 min (Lee et al., 2008). This process has been suggested in cases where exogenous, monomeric α-synuclein can enter the cell regardless of the application of endocytic inhibitors such as low temperature or dynamin inhibition (Lee et al., 2008; Steiner et al., 2011).

#### 2.5.3. Trafficking

The processing of α-synuclein in the cell is a vital determinant of its toxicity and pathogenesis (Flavin et al., 2017). Studies in induced pluripotent stem cells differentiated into dopaminergic neurons found that α-synuclein was trafficked a considerable distance in the cell, including through the soma and cellular projections and that fusion of ruptured vesicles due to α-synuclein aggregation led to the formation of large cytoplasmic inclusions (Flavin et al., 2017).

##### 2.5.3.1. Endocytic vesicle trafficking

Extracellular α-synuclein is primarily trafficked through the endocytic pathway where it is delivered to the lysosome for clearance (Figure 3; Delenclos et al., 2017; Lee et al., 2018). Fibrils move through endosomal compartments after internalization (Lee et al., 2008). Fluorescent imaging has shown α-synuclein species with within early endosomal antigen 1 (EEA1) positive vesicles (a Rab5a effector protein) and lysosomal associated membrane protein 1 (LAMP1) positive lysosomes (Masaracchia et al., 2018; Fakhree et al., 2021). Screening methods have found functional interaction between α-synuclein and Rab proteins (Dalfó et al., 2004) and α-synuclein has been seen to disrupt Rab homeostasis (Gitler et al., 2008).

Importantly, it is the evasion of the endolysosomal pathway that is vital for pathogenesis of α-synuclein (Rodriguez et al., 2018). It has been proposed that α-synuclein is capable of inducing vesicle rupture in endosomes and lysosomes to release into the cytoplasm (Flavin et al., 2017). Large fibrillar species then act as a template to seed endogenous monomers to form large proteinaceous complexes and lead to cell death (Figure 3; Freeman et al., 2013).

##### 2.5.3.2. Microtubule trafficking

Axonal trafficking of neuronal proteins is primarily mediated by networks of microtubules from the cell body to the synaptic bouton and is fundamental for neuronal homeostasis and survival (Carnwath et al., 2018; Guedes-Dias and Holzbaur, 2019). As α-synuclein primarily localizes to the pre-synaptic terminal, it has been demonstrated to undergo axonal transport via the slow component, driven by the microtubule cytoskeleton and motor proteins kinesin and dynein (Utton et al., 2005; Carnwath et al., 2018). *In vivo* and *in vitro* studies have found α-synuclein to co-localize with microtubules and can influence the stability of microtubules (Lee et al., 2006; Prots et al., 2013; Carnwath et al., 2018).

Mechanisms of neurodegeneration can include the retreating of axons into the cell soma, a process thought to be caused by microtubule depolymerization (Carnwath et al., 2018). In sporadic PD brains, a decline in motor proteins was correlated with increased α-synuclein aggregates (Chu et al., 2012; Prots et al., 2013). Chronic α-synuclein overexpression in primary hippocampal neurons impaired neurite elongation due to interruption of tubulin polymerization (Koch et al., 2015). Therefore, interaction between pathogenic α-synuclein and microtubules could lead to neurodegeneration.

### 2.6. Clearance mechanisms

α-synuclein clearance mechanisms have been elucidated for the purpose of therapeutic intervention. Enhancing degradation of the higher molecular weight species of α-synuclein has the potential to reduce its pathology (Stefanis, 2012; Dikic, 2017). There are two major protein degradation machineries within the cell, the ubiquitin proteasome system and the autophagosome lysosome pathway (Bennett et al., 1999; Lilienbaum, 2013; Dikic, 2017). There is an intricate crosstalk between these pathways engaged in the processing of α-synuclein (Lilienbaum, 2013). Several studies have demonstrated preferential degradation by each system, demonstrating it is cell and model dependent (Mattos et al., 2020). For example, overexpression models can impair the activity of the ubiquitin proteasome system and the autophagy lysosomal pathway and accelerate aggregation and toxicity (Mattos et al., 2020). The ubiquitin proteasome system is suggested to play a prominent role in degrading small, soluble α-synuclein assemblies in healthy and functional systems, however, autophagic activity is required for larger α-synuclein assemblies such as fibrillar species or mutants (Bennett et al., 1999; Mattos et al., 2020).

#### 2.6.1. Autophagolysosomal pathway

Following internalization, α-synuclein fibrils are trafficked to late endosomal compartments and into lysosomes (Figure 3; Vogiatzi et al., 2008; Dilsizoglu Senol et al., 2021). Lysosomal inhibition has been shown to cause accumulation of α-synuclein in early and late endosomes, suggesting an important role for the lysosomal pathway in clearance of α-synuclein (Vogiatzi et al., 2008; Cunningham and Moore, 2020). Furthermore, extracellular aggregated α-synuclein alters lysosomal morphology and function (Cunningham and Moore, 2020).

The autophagolysosomal pathway comprises several catabolic processes that converge at the lysosome and are three main processes: Macroautophagy, chaperone-mediated autophagy, and microautophagy (Konno et al., 2012; Parzych and Klionsky, 2014). Macroautophagy begins with the engulfment of proteins in autophagosomes and targeted to lysosomes for degradation. The majority of large protein aggregates are degraded via macroautophagy (Parzych and Klionsky, 2014). Inhibitors of macroautophagy have seen a build-up of α-synuclein monomeric and fibrillar species *in vitro* (Konno et al., 2012). This suggests a role for macroautophagy in the degradation of many conformations of α-synuclein (Konno et al., 2012). Chaperone mediated autophagy involves the selective targeting of substrates to the lysosome via Hsc70 that recognize cargo with the KFERQ-motif (Kaushik and Cuervo, 2018). Chaperone mediated autophagy is involved in the processing of α-synuclein as *in vitro* studies have shown α-synuclein contains the motif and directly interacts with LAMP2a and Hsc70 (Kaushik and Cuervo, 2018). Mutations to the KFERQ-motif abolished lysosomal degradation of α-synuclein (Wildburger et al., 2020). Several acidic proteases are believed to breakdown α-synuclein in lysosomes, such as cathepsins and glucocerebrosidase (Wildburger et al., 2020). Microautophagy is the direct uptake of cytoplasm into lysosomes, which can be in bulk or selective fashion (Lee et al., 2013). It is unclear in research as to whether α-synuclein is cleared via microautophagy, where macroautophagy and chaperone-mediated autophagy appear to be the primary degradation pathways of pathogenic α-synuclein (Lee et al., 2013; Fonseca et al., 2015).

### 2.7. Pathogenic propagation

Cell-to-cell spreading of α-synuclein has been implicated in PD by findings in a variety of cell and animal models (Masuda-Suzukake et al., 2014). Unilateral injection of wild-type mice with recombinant α-synuclein fibrils demonstrated after 1 month that α-synuclein could propagate along neural circuits and deposit in various parts of the brain (Masuda-Suzukake et al., 2014). As discussed, non-motor symptoms of PD, including disturbances in sensory and autonomic function, pre-date motor symptoms by up to 20 years (Kalia and Lang, 2015). Such features can be speculated to be associated with α-synuclein deposition in olfactory bulb and enteric nervous system (Steiner et al., 2018). Therefore, α-synuclein has been identified as a prion-like protein as it assembles acts as seeds for further aggregation (Steiner et al., 2018). It is important to understand the routes of propagation and to identify molecular and cellular targets for therapeutic prevention. With the understanding of how the protein is internalized, trafficked and cleared, it can then be deduced and investigated as to how pathogenic propagation occurs.

Evasion of the endocytic pathway, autophagolysosomal and ubiquitin proteasome system allows for the protein to administer toxicity within the cell (Steiner et al., 2018). Evasion of the endolysosomal pathway via vesicle rupture is one mechanism of α-synuclein toxicity, but how the protein propagates cell-to-cell is complex and entails several multifaceted mechanisms (Flavin et al., 2017). Interestingly, in co-culture experiments, aggregated α-synuclein species exhibit stronger accumulation in recipient cells and are more efficiently internalized than non-toxic species (Bae et al., 2015). This demonstrates that toxic species of α-synuclein have an affinity to spread and infect neighboring cells.

#### 2.7.1. Seeding of endogenous molecules

Oligomeric and fibrillar species of α-synuclein can spread cell-to-cell, evade protein degradation systems and deposit within the cytoplasm and other organelles in the cell (such as the mitochondria) (Fonseca et al., 2015). Here, these toxic species can seed endogenous protein to form larger aggregates leading to cellular toxicity and eventual death (Fonseca et al., 2015). Intracellular α-synuclein does not aggregate under healthy conditions as it is limited by the number of active nucleation sites within the cytoplasm (Wood et al., 1999). Exogenously added α-synuclein fibrils can alter this by acting as seeds for aggregation by presenting nucleation sites (Luk et al., 2009). Further, increased concentrations of α-synuclein also enhances the kinetics of fibrillization, as determined by genetic studies (Conway et al., 1998). Fibrillar species act as exogenous seeds, however, monomers do not seed the formation of aggregates in neuronal and non-neuronal cell models and *in vivo* (Luk et al., 2009). Oligomeric species are also believed to have the capacity to template aggregation, however, some studies dispute this as intracellular aggregation does not occur upon exposure to soluble species of α-synuclein (Waxman and Giasson, 2009). Inhibition/reduction of the seeding properties of α-synuclein has been used for drug screening *in situ* as this is a vital pathogenic pathway for the protein (Ardah et al., 2020).

#### 2.7.2. Tunneling nanotubules

α-synuclein can be secreted through tunneling nanotubules (TnTs) to neighboring cells providing a direct path for the spreading of pathology (Abounit et al., 2016). TnTs are actin-based membrane channels that directly connect cells (Abounit et al., 2016). Mono- and co-culture experiments have been used to directly visualize TnTs using microscopy (Abounit et al., 2016; Dieriks et al., 2017; Valdinocci et al., 2021). TnT transfer has been demonstrated in a variety of cellular models and α-synuclein can be involved with lysosomal and mitochondrial membranes within the TNTs (Abounit et al., 2016; Valdinocci et al., 2021). Studies have found that the spread of α-synuclein through TnTs is not cell type specific and has been demonstrated in both neuronal and non-neuronal models (Abounit et al., 2016; Valdinocci et al., 2021).

Intercellular exchange of mitochondria has been previously defined previously for respiration rescue of neighboring cells (Wang et al., 2019). A recent study has found that aggregated α-synuclein is associated with mitochondria within TnTs in KCl-treated SH-SY5Y cells (Valdinocci et al., 2021). Utilizing fluorescence microscopy, TnT structures were found to contain α-synuclein bound TOM-20 positive mitochondria (Valdinocci et al., 2021). The same study demonstrated similar results for human astrocytes in monoculture and co-culture (Valdinocci et al., 2021). The evasion of cellular degradation systems by α-synuclein via TnT structures can therefore occur within lysosomes or mitochondrion which facilitates cell-to-cell spread of the protein (Abounit et al., 2016; Valdinocci et al., 2021).

#### 2.7.3. Exocytosis

A cellular secretory mechanism known as exocytosis utilizes the release of cargo in the form of 30–150 nm vesicles known as exosomes (Johnstone et al., 1987; Doyle and Wang, 2019). Exosomes are formed from multivesicular bodies that can fuse with the plasma membrane and release the contents to the extracellular environment (Johnstone et al., 1987; Doyle and Wang, 2019). One proposed pathway of α-synuclein exocytosis involves secretory autophagosomes that are in the process of α-synuclein degradation fusing with multi vesicular bodies to form amphisomes (Danzer et al., 2012; Rodriguez et al., 2018). These secretory vesicles can fuse with the plasma membrane and release α-synuclein into the extracellular space (Danzer et al., 2012; Rodriguez et al., 2018). *In vitro* studies have shown that α-synuclein oligomers can utilize exosomes and are then preferentially endocytosed by neighboring cells (Danzer et al., 2012). However, this has still not been well defined in literature as to the precise mechanism that α-synuclein uses exosomes for release.

## 3. Proposed strategies for therapies targeting the biology of α-synuclein

Recent therapeutic strategies for treatment of PD have aimed at inhibiting the pathology of α-synuclein with varying success. Researchers have developed drugs to reduce α-synuclein production, inhibit α-synuclein aggregation, inhibit α-synuclein uptake, and enhance the clearance of α-synuclein. These inhibitors have been screened through a variety of methodologies including Thioflavin-T assay, electron microscopy, *in vitro* and *in vivo* models. The wide variety of screening techniques and previously discovered small molecule inhibitors of α-synuclein provide a valuable insight into how drug screening can be adapted and improved.

### 3.1. Reducing α-synuclein production (RNAi)

One model of reducing the toxicity of α-synuclein was to reduce the production of the protein using small interfering RNA (RNAi) (Fields et al., 2019). *In vivo* models have shown that delivery of naked small interfering RNA or lentiviral-mediated RNAi for α-synuclein silence in the rodent brain can reduce α-synuclein levels and prevent neurodegeneration (Uehara et al., 2019). *In vitro* and *in vivo* experiments using amido-bridged nucleic acid-modified antisense oligonucleotide resulted in decreased mRNA and improved motor deficits in PD mouse model (Uehara et al., 2019). However, further studies have shown that the reduction of α-synuclein levels can cause degenerative outcomes therefore this is not the best model for treatment of PD (Fields et al., 2019).

### 3.2. Inhibitors of aggregation

Inhibition of α-synuclein aggregation is the newest model of treatment options for people living with PD and the eventual prevention of neurodegeneration (Fields et al., 2019). Furthermore, identification of aggregation inhibitors in a variety of screening models has been a priority in PD research. A wide variety of small molecules have been found to inhibit α-synuclein aggregation (Xu et al., 2019). Antibiotics, curcuminoids, flavanols, quinones, pyrazines, bromotyrosine derivatives, and polyoxygenated sterols are all small molecules found to have inhibited the aggregation of α-synuclein (Kobayashi et al., 2006; Singh et al., 2016; Yedlapudi et al., 2016; Javed et al., 2018; Heras-Garvin et al., 2019; Jennings et al., 2020; Prebble et al., 2021, 2022). However, issues arise when the drugs are tested *in vitro* and *in vivo.* Compounds are often quickly oxidized and therefore unstable, cannot pass the blood brain barrier or induce cytotoxicity to cells. Therefore, investigating small molecule inhibition of α-synuclein has been demonstrated in trafficking models of the protein.

### 3.3. Inhibitors of uptake

Interfering with the pathology of α-synuclein has evolved from prevention/inhibition of aggregation to interference of the prion-like spread of the protein (Fields et al., 2019). Therefore, blocking the major uptake receptors has also been used in research efforts, however, is not yet translated to medicine (Fields et al., 2019).

#### 3.3.1. Dynamin inhibitors

Use of the clathrin-mediated endocytosis blocker dynasore is widely used to inhibit uptake in cellular models (Oh et al., 2016). In donor-acceptor co-culture methods that demonstrate the transfer of α-synuclein from donor cells to acceptor cells; transmission was infrequent with dynasore treatment (Oh et al., 2016; Masaracchia et al., 2018). As clathrin-mediated endocytosis is a widely used process majority of mammalian cells it is not feasible to completely inhibit the pathway to reduce α-synuclein pathology (Konno et al., 2012). Therefore, researchers sourced a pharmacological intervention that disrupts dynamin GTPases but does not completely abolish endocytosis (Konno et al., 2012). With the administration of sertraline, an antidepressant, cells significantly decreased the internalization and translocation of α-synuclein (Konno et al., 2012). As this is a widely used drug, the side effects can be well managed and is previously clinically tested (Konno et al., 2012).

#### 3.3.2. LAG3 antibody

LAG3 is a cell-surface protein that functions as an immune receptor directly involved in the endocytosis of extracellular fibrillar α-synuclein (Mao et al., 2016). LAG3-directed antibodies and silencing of the gene can significantly reduce α-synuclein aggregate transmission and toxicity as quantified by levels of phosphorylated protein at serine 129 (Mao et al., 2016). *In vivo*, knock down of LAG3 also decreased PFF-induced death of dopaminergic neurons and associated behavioral and motor deficits (Mao et al., 2016). Pharmaceutical agents are being developed to find LAG3 blockers that do not induce cytotoxicity (Mao et al., 2016).

#### 3.3.3. Soluble heparin

Another mode of endocytosis for α-synuclein is via binding to HSPGs (Holmes et al., 2013). Known inhibitors of these glycoproteins are soluble heparin, heparinase, and chloral hydrate (Holmes et al., 2013; Ihse et al., 2017; Fields et al., 2019). *In vitro*, administration of these compounds interferes with HSPGs and decreases uptake of pathogenic α-synuclein (Holmes et al., 2013; Fields et al., 2019). However, blocking this pathway can interfere with other vital cellular processes and continues to be investigated for toxicity and reproducibility (Fields et al., 2019).

### 3.4. Enhancers of clearance

As discussed, new ways of inhibiting the pathogenesis of α-synuclein are a wide topic of research. Enhancing the clearance pathways to degrade α-synuclein is also being studied (Fields et al., 2019).

#### 3.4.1. Rapamycin

The role of the autophagolysosomal pathway in PD is well developed and researchers have sought to enhance clearance of misfolded α-synuclein (Oh et al., 2017). The mammalian target of rapamycin (mTOR) is a conserved serine/threonine protein kinase that plays a role in the process and termination of autophagy (Kim and Guan, 2015). Rapamycin is a direct inhibitor of mTOR and a potent inducer of autophagy (Kim and Guan, 2015). mTOR expression levels increase in overexpression models of α-synuclein (Ramalingam et al., 2019). Rapamycin can reverse the increased mTOR activity induced by α-synuclein and assist in clearance of α-synuclein (Ramalingam et al., 2019). Rapamycin was shown to directly reduce α-synuclein accumulation in PC-12 cell model by inhibiting the termination of autophagy (Sarkar et al., 2005). Rapamycin has also been used *in vivo* where it improved the motor function in A53T transgenic mice (Bai et al., 2015). However, rapamycin has been seen to cause immunosuppression and is therefore not an ideal drug candidate.

#### 3.4.2. Trehalose

Disaccharide trehalose induces autophagy and has displayed therapeutic effects *in vitro and in vivo* models of neurodegeneration (Hoffmann et al., 2019; Pupyshev et al., 2019). Trehalose has the properties of a chaperone and can induce chaperone-mediated autophagy independent of mTOR autophagy (Pupyshev et al., 2019). Trehalose enhances lysosomal function and biogenesis resulting in clearance of α-synuclein (Hoffmann et al., 2019). Trehalose is controversial as may not protect cultured neurons against toxicity and therefore has not been widely accepted (Lee et al., 2018).

#### 3.4.3. AR7

Pharmacological manipulation of the chaperone-mediated autophagy pathway using AR7, a retinoic acid receptor alpha antagonist in mutant fibroblasts restores lysosomal function (Ho et al., 2020). Attenuated progressive accumulation of α-synuclein oligomers (Ho et al., 2020). However, the compound is unstable and could limit medical use. Therefore, chemists have sought to synthesize derivatives of AR7 that have shown strong aggregation inhibition *in situ* (Ho et al., 2020).

### 3.5. Enhancers of trafficking

Although not as widely studied as the enhancement of clearance of toxic α-synuclein species, enhancement of trafficking has also been examined to reduce pathogenesis (Gitler et al., 2008; Dinter et al., 2016).

#### 3.5.1. Overexpression of Rab proteins

*In vivo* and *in vitro* models have shown that overexpression of Rab1, Rab3a, Rab7, and Rab8a have provided substantial rescue of α-synuclein-induced pathogenesis (Gitler et al., 2008; Dinter et al., 2016). These studies together suggest an important role of the trafficking process of α-synuclein, whereby overexpressing proteins in this pathway could reduce pathology (Gitler et al., 2008; Dinter et al., 2016). However, overexpression of Rab proteins are also seen in cancers and other neurodegenerative diseases, such as Alzheimer’s disease, debating this as a therapeutic option for PD (Li, 2011; Jordan et al., 2022).

At least 5 α-synuclein targeted programs in phase 1 or 2 of clinical testing. However, due to a lack of biomarkers, this can lead to uninformed results (Brundin et al., 2017). It is still unclear what molecular species of α-synuclein is best to target, as this could provide different pathogenicity between patients (Brundin et al., 2017).

## 4. Concluding remarks

Parkinson’s disease is a complex and multifactorial neurological disorder that can be characterized by misfolded α-synuclein in proteinaceous inclusions. Underlying genetic risk factors for disease have elucidated the cellular trafficking events that may lead to disease state, and the importance of α-synuclein trafficking for pathogenesis. Therefore, there has been a great need for relevant models for the uptake, processing and clearance of α-synuclein. This review has explored the different forms α-synuclein can take and thus different cellular pathways that process the variety of conformations of α-synuclein. Moreover, the analysis of these different uptake and clearance systems highlights the importance for further understanding of α-synuclein trafficking and its biology. It is also clear that more targeted strategies to reduce the pathogenesis of α-synuclein are needed for the treatment of PD without damaging the vital trafficking systems of the cell.

## Author contributions

JS, AS, and GM involved in conceptualization, writing, and editing the final manuscript. All authors that contributed to the article and approved the submitted version.
